# Designing Multipolar Resonances in Dielectric Metamaterials

**DOI:** 10.1038/srep38487

**Published:** 2016-12-08

**Authors:** Nikita A. Butakov, Jon A. Schuller

**Affiliations:** 1 University of California, Santa Barbara, Department of Electrical & Computer Engineering, USA.

## Abstract

Dielectric resonators form the building blocks of nano-scale optical antennas and metamaterials. Due to their multipolar resonant response and low intrinsic losses they offer design flexibility and high-efficiency performance. These resonators are typically described in terms of a spherical harmonic decomposition with Mie theory. In experimental realizations however, a departure from spherical symmetry and the use of high-index substrates leads to new features appearing in the multipolar response. To clarify this behavior, we present a systematic experimental and numerical characterization of Silicon disk resonators. We demonstrate that for disk resonators on low-index quartz substrates, the electric and magnetic dipole modes are easily identifiable across a wide range of aspect-ratios, but that higher order peaks cannot be unambiguously associated with any specific multipolar mode. On high-index Silicon substrates, even the fundamental dipole modes do not have a clear association. When arranged into arrays, resonances are shifted and pronounced preferential forward and backward scattering conditions appear, which are not as apparent in individual resonators and may be associated with interference between multipolar modes. These findings present new opportunities for engineering the multipolar scattering response of dielectric optical antennas and metamaterials, and provide a strategy for designing nano-optical components with unique functionalities.

Dielectric resonators, first developed as radio-frequency antenna elements^1^, are now being used to develop high-performance nano-optical components. In particular, dielectric Mie resonators support a complete set of multipolar harmonics^2^, including magnetic dipole and quadrupolar modes. Due to their low intrinsic losses^3^,^4^, as compared to metal-based plasmonic antennas, dielectric resonators make attractive building blocks for metamaterial and metasurface based technologies. Recent demonstrations include ultra-thin lenses^5^,^6^,^7^,^8^,^9^,^10^,^11^, blazed gratings^12^,^13^, vector beam generators^14^,^15^, beam shaping^16^, fluorescence and nonlinearity enhancement^17^,^18^,^19^, optical antennas^20^,^21^,^22^,^23^,^24^,^25^, and reconfigurable metamaterials^26^,^27^.

A common dielectric metasurface building block is a high-index sphere with a scattering response that can be decomposed into spherical harmonics (Fig. 1a) through Mie Theory^28^. Practical implementations favor easier to fabricate disk-shaped resonators, (Fig. 1b), which also provide additional design parameters: the aspect-ratio (AR) and substrate refractive index. When the aspect-ratio and substrate refractive index are close to unity, these disk-resonators may be considered a small perturbation^29^,^30^ of sphere resonators. However, when the aspect-ratio of the disk becomes large, or when a high-index substrate is introduced, the breaking of spherical symmetry introduces new features into the multipolar modes. It has been shown that for metallic nanostructures the presence of a dielectric substrate can couple and hybridize the resonator’s plasmonic modes^31^,^32^. When particles are arranged into arrays, inter-particle interference further modifies the optical response.

In this work, we systematically measure the dipolar and quadrupolar resonances of individual disks and disk arrays with varying aspect-ratio and substrate index. With standard cleanroom processes we fabricate Silicon disk resonators (Fig. 1c) and determine their resonance wavelengths through FTIR reflection and transmission measurements. With electromagnetics simulations, we then decompose the internal fields of the resonators into Cartesian multipole moments (Equations 1, 2, 3, 4), which include toroidal contributions (Equations 5 and 6). We neglect higher order mean-square radii correction terms, which represent a minor perturbation^33^. The approximate partial scattering-cross-sections (Equations 7, 8, 9, 10) are then calculated to track the dispersion of individual multipolar modes^34^,^35^. In doing so, we map the dispersion of multipolar resonances with respect to aspect ratio, demonstrate unique features of the multipolar response such as overlapping electric and magnetic modes^36^ and a frequency-shifted response in disk arrays, as compared to single resonators.


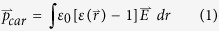



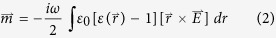



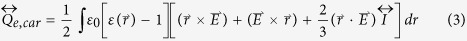



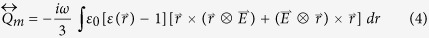



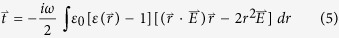



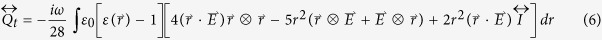



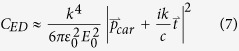



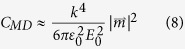



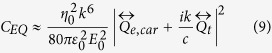



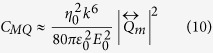


## Individual Silicon Disks on Low-Index Substrates

**Figure 2:**
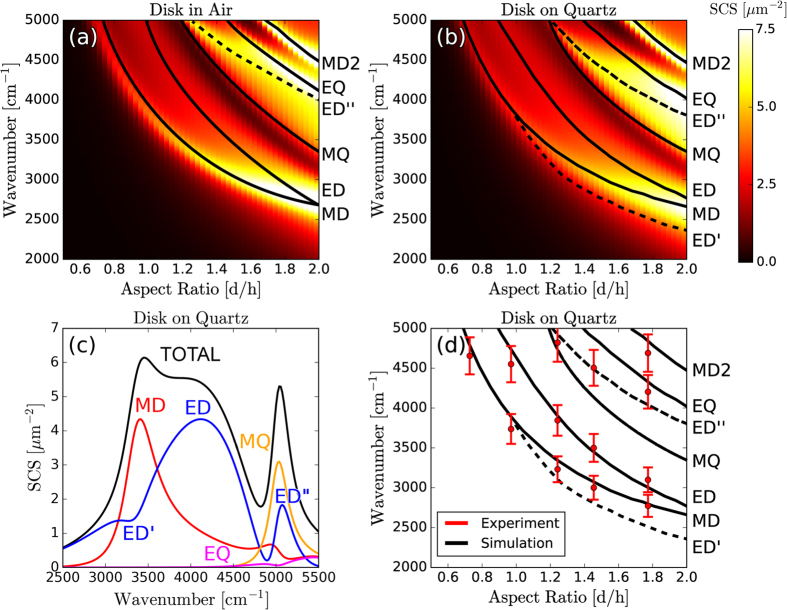
Individual Resonators on Low-Index Quartz Substrates. The simulated scattering-cross-section of an individual Silicon resonator with height h = 660 nm, as a function of the aspect ratio (diameter/height) with (**a**) no substrate, and (**b**) a low-index quartz substrate. (**c**) A line-cut of the scattering cross section given in (**b**) at an aspect ratio AR = 1.2, with individual multipole contributions. (**d**) The experimentally measured transmission dips of Silicon resonators on a quartz substrate, compared to the simulated dispersion of the multipolar resonances. The height of each resonator is fixed at h = 660 nm and the aspect-ratio is varied by changing the diameter. Simulation and experimental details are in the Methods and Supplementary Material.

In Fig. 2 we plot the simulated scattering-cross-section of varying aspect ratio (diameter/height) Silicon disk resonators in air (Fig. 2a), and on a quartz substrate (Fig. 2b). Resonances in the multipolar moments are determined by decomposing the fields internal to the disk according to equations 1, 2, 3, 4, 5, 6, 7, 8. Black lines are local maxima in the modal decompositions of the *total* multipole moments (i.e. including toroidal contributions). Consider Fig. 2c, the scattering cross-section of an AR = 1.2 disk on quartz (equivalent to a line-cut taken vertically through Fig. 2b). The breaking of spherical symmetry is evident in an additional peak in the electric dipole moment near 5000 cm^−1^, labeled ED”. There is also an additional, substrate-induced peak in the electric dipole moment at low frequencies, labeled ED’. This local peak becomes even more prevalent for disks on high-index substrates, and is discussed in more detail in the subsequent section. These ED, ED’, and ED” resonances are similar in that they all represent local maxima in the electric dipole moment. However the ED mode has a clear correspondence in spherical particles, whereas the ED’ and ED” resonances only appear due to symmetry breaking. To highlight this difference, they are represented as dashed lines in Fig. 2a and b. Similar to spheres, the modes disperse to lower frequencies with increasing radius. However, dispersion of the fundamental ED and MD modes flattens as the aspect ratio increases towards 2.0. Similar phenomena were recently observed in 0-D disk and 1-D rectangular Mie resonators of varying widths^37^,^38^. Additionally, the ED and MD resonances approach each other and overlap at an aspect ratio of ~2, a phenomenon that is particularly important for the design of transmissive metasurfaces^39^,^40^,^41^.

In Fig. 2d we plot experimentally measured dips (see Methods for optical characterization details) in the experimental transmission spectra (see supplementary material for experimental spectral line-shapes) of single resonators, along with their simulated dispersion curves. The lowest order MD and ED modes are easily identified and track the calculated dispersion of multipolar moments. However, higher order modes (i.e. ED”, MQ, and EQ resonances) do not appear as distinguishable features in the experimental spectra. At high frequencies, the observed transmission dips most closely track the ED” resonance, as expected from simulations (Fig. 2b). The interference of multipole resonances with each other, and with reflections off the substrate, leads to a broad scattering resonance whose maximum cannot be attributed to any individual multipole moment.

## Individual Silicon Disks on High-Index Substrates

**Figure 3:**
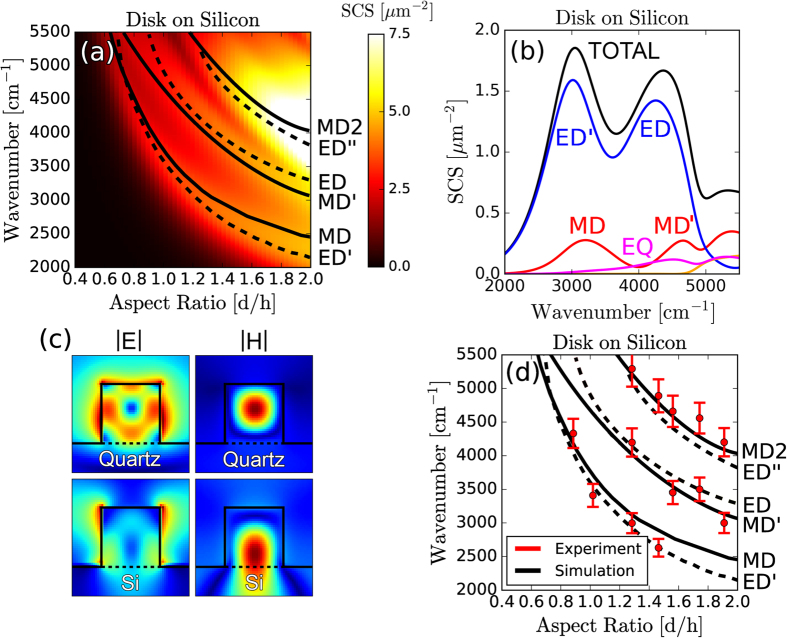
Individual Resonators on High-Index Silicon Substrates. (**a**) The simulated scattering-cross-section of an individual Silicon resonator, with height h = 660 nm, as a function of the aspect ratio, on a high-index Silicon substrate. (**b**) A line-cut of the decomposed scattering cross section given in (**a**) at an aspect ratio AR = 1.2. (**c**) The local electric and magnetic field plots of Silicon resonators, with AR = 1, on Quartz and Silicon substrates. (**d**) The experimentally measured reflection peaks of individual Silicon resonators on a Silicon substrate, compared to the simulated dispersion of the multipolar resonances. The height of each resonator is fixed at h = 660 nm and the aspect-ratio is varied by changing the diameter. Simulation and experimental details are in the Methods and Supplementary Material.

The substrate effects seen for resonators on quartz (n≈1.45) become far more prominent for resonators on higher index substrates. In Fig. 3a we plot the simulated scattering-cross-section of an individual Silicon resonator, on a high-index Silicon substrate (n≈3.4), as a function of the disk AR. Compared to the scattering response of identical resonators on low-index substrates, linewidths are substantially broadened and the fundamental ED and MD modes shift away from each other so that they no longer overlap. Additionally, each of the three lowest order scattering resonances exhibits substantial mode hybridization. Consider Fig. 3b, which shows a line-cut of Fig. 3a at AR = 1.2. The ED moment dominates over the competing multipoles. The origin of this behavior can be understood through examination of electromagnetic intensity plots. In Fig. 3c we plot the local electric and magnetic field intensities for the lowest order scattering resonance of Silicon disks on Quartz (top) and Silicon substrates (bottom). For the quartz substrate, a resonant magnetic field is positioned in the center of the disk. The electric fields are zero at the center and exhibit an approximately circular band of high intensity internal to the disk. These field patterns are quite similar to the MD modes of spheres, and the multipolar decomposition is dominated by the MD moment. On the high-index substrate, however, the field patterns are significantly altered. The peak magnetic field is pushed closer to the substrate and the dominant electric field intensity maxima are external to the disk. The multipolar decomposition is dominated instead by the ED moment^42^. It is important to note that multipolar decompositions are not unique, and will vary with choice of origin. We choose the disk center to facilitate comparison across different geometries. In any case, there is a significant perturbation of field profiles and multipolar moments for disks on high index substrates, and further work is needed to understand how this may affect, for example, the homogenized metamaterial response in such geometries. In Fig. 3d we compare the location of resonances in the multipolar moments with experimental measurements of reflection minima. We observe the dispersion of three clear sets of scattering peaks. In simulations however, each of these three clear curves does not have an obvious correspondence to a single multipolar mode. Unlike the previous case of resonators on a Quartz substrate, here even the fundamental mode appears to track the combined response of the MD and ED decomposition.

## Collective Interference in Silicon Disk Arrays

**Figure 4:**
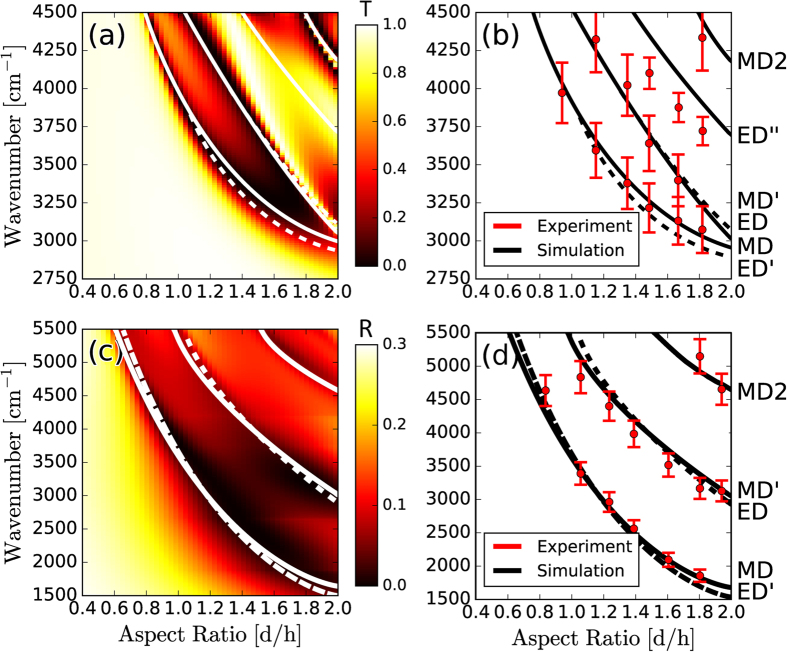
Disk Resonators Arrays on Low- and High-Index Substrates. (**a**) The simulated transmission spectra, as a function of disk aspect ratio, for an array of Silicon resonators on a Quartz substrate with period a = 1.55 μm. (**b**) The experimentally identified modal peaks and the simulated dispersion curves. (**c**) The simulated reflection spectra, as a function of disk aspect ratio, for an array of Silicon resonators on a Silicon substrate with period a = 1.55 μm. (**d**) The experimentally identified modal peaks and the simulated dispersion curves.

In arrays, inter-particle interference modifies the collective scattering response^43^,^44^,^45^,^46^. In Fig. 4a we plot the simulated transmission spectra of Silicon disk resonators, on a low-index quartz substrate, with sub-wavelength periodicity a = 1.55 μm and varying AR. Scattering resonances of disks now appear as minima in the transmission spectra. We see that the lowest order ED and MD modes are relatively narrow, as compared to the broader scattering response of individual resonators in Fig. 2b, and unlike individual resonators, can be independently resolved over a larger range of aspect-ratios. Due to near-field coupling, the fundamental MD mode is blue-shifted relative to the individual resonators in Fig. 2b. A narrow transmission peak appears at frequencies just above the ED mode due to the Kerker condition, in which the ED and MD modes interfere in-phase and enhance forward scattering. Although this Kerker condition is fulfilled in individual resonators as well^47^,^48^,^49^, it is much more apparent in the arrays. At frequencies above the Kerker condition, features in simulated (Fig. 4a) or experimental (Fig. 4b) transmission spectra can no longer be ascribed to individual multipolar resonances. In particular, the third set of experimentally observed peaks in Fig. 4b is substantially red-shifted relative to the peak of the ED” resonance. We speculate that this is due to the fulfillment of a preferentially backward scattering condition, or anti-Kerker condition, in which multipoles constructively interfere in the backward direction. Similar effects were recently observed in single dielectric spheres^50^. Furthermore, higher order quadrupole moments appear to be significantly suppressed, even at high frequencies near the MD2 peak.

On high index substrates (Fig. 4c,d) the relationship between scattering and transmission is inverted—scattering resonances are correlated with peaks in transmission, or equivalently dips in reflection (see supplementary for experimental spectral line shapes). Due to its higher refractive index, and thus higher optical density of states, most of the scattered light is directed into the substrate^51^. Similar to individual resonators, we see that disk arrays on high-index substrates exhibit substantially broader ED and MD resonances compared to low-index substrates. Here however, in contrast to low-index Quartz substrates, the fundamental MD mode is red-shifted. We speculate that high-index substrates effectively reduce near-field coupling, due to reduced wavelengths, and that radiative interference leads to prominent red-shifting^52^.

## Conclusion

In conclusion, we have elucidated the dependence of the multipolar scattering properties of dielectric disk-resonators, as a function of the disk aspect ratio and substrate refractive index. We demonstrate that changing the disk aspect-ratios or substrate refractive index can lead to overlapping electric and magnetic multipolar modes. For individual disks on Quartz substrates, dipolar modes are easily identified in numerical multipolar decompositions, but at higher frequencies broad scattering peaks cannot be easily attributed to any specific higher order multipole resonance. On high-index Silicon substrates, internal field patterns are significantly altered, and even the fundamental dipole modes cannot be unambiguously associated with an electric or magnetic type decomposition. When arranged into arrays, new effects arise due to interference between multipolar modes. On Quartz substrates the fundamental magnetic dipole mode is blue-shifted, and at higher frequencies there exist preferentially forward-scattering Kerker and preferentially backward-scattering anti-Kerker resonances. On high-index substrates however, the fundamental MD mode is strongly red-shifted, and highly directional scattering resonances are not apparent. These findings provide new insights into designing dielectric optical antennas, and will serve as guidelines for engineering metamaterials with novel functionalities.

## Methods

### Fabrication and Characterization

Prior to processing, substrates were ultrasonicated and cleaned with acetone, isopropanol, ‘nanostrip’ solution to remove organic contaminants, and HF to remove native oxide. Amorphous Silicon was deposited onto fused Quartz substrates with an Advanced Vacuum PECVD by decomposing silane with an electron plasma (650 mT, 2% SiH_4_ at 1500 sccm, high-frequency power 30 W, chuck temperature 300 C). Although there are differences in the optical constants of crystalline vs. amorphous Silicon in the visible spectrum, for the mid-infrared range examined in this work the difference in refractive index is negligible.

To compensate for stress-induced curvature across the sample, a film of SiO_2_ was deposited on the back-side. In preparation for deep ultraviolet photolithography with an ASML PAS 5500/300 DUV Stepper, an anti-reflective coating (AR15) and negative-type photoresist (UVN-2300) were spun onto the samples. The pattern was exposed with annular-type illumination with NA = 0.63, outer sigma = 0.8, inner sigma = 0.5, exposure dose = 109 mJ/cm^2^, and a focus offset −0.15 um, which enabled us to reliably achieve sub-150 nm feature sizes. After exposure, wafers were post-baked for 60 seconds at 105 C and develop with AZ300MIF for 30 seconds. Leftover AR and PR was removed in an O_2_ barrel asher for 30 seconds at 300 mT and 100 W.

Samples were etched with a deep reactive-ion-etching process using a PlasmaTherm 770 SLR system. Prior to etching the chamber was plasma cleaned for 30 minutes at 30 mT, 0 W substrate bias, 825 W ICP power, 20 sccm O_2_, and 10 sccm Ar. Etching was performed at 19 mT, 15 W substrate bias, 825 W ICP power, 26 sccm SF_6_, 54 sccm C_4_F_8_, 20 sccm Ar, with He backside cooling. Afterwards the photoresist was removed with an O_2_ plasma in a Gasonics ashing system at >350 C.

The reflection and transmission spectra of the Silicon disk resonators were measured with a Bruker Vertex FTIR and Hyperion Microscope. Simplified diagrams of the optical path in the reflection and transmission configurations are given in the supplementary material. Individual isolated resonators were identified and focused using visible light in the microscope. The infrared spectra were then measured with the MCT detector.

### Simulation

All electromagnetic simulations were performed with the Lumerical FDTD Solver. Individual disk resonators were simulated with PML boundary conditions and boundaries at least 5 um away from the resonator. The resonator was excited with a total-field-scattered-field source. The total scattering cross section was measured using the built-in ‘cross-section’ analysis object. The forward and backward scattering cross sections are the field power transmitted through the forward and backward hemispheres of the ‘cross-section’ monitor respectively. The partial scattering cross sections were determined by performing a multipole decomposition based on the internal electric fields of the particle. Expressions for the multipolar moments were integrated across the volume of the disk, and the corresponding partial scattering cross section was subsequently calculated. The origin was taken to be at the center of the disk for all calculations. The Silicon and Quartz optical constants were modelled using a Lumerical fit to data collected by Palik. Convergence tests indicated a mesh size of less than 7.5 nm inside the disk resonator and a simulation time of at least 4,500 fs was needed.

Disk resonator arrays were simulated with PML boundary conditions in the z direction, and periodic boundary conditions along the x and y directions, and with an anti-symmetric condition applied along the x-direction and symmetric condition applied along the y-direction to improve computational efficiency. The disks were excited with a plane-wave source. The reflection and transmission scattering parameters were computed with the built-in ‘s_params’ analysis object.

## Additional Information

**How to cite this article**: Butakov, N. A. and Schuller, J. A. Designing Multipolar Resonances in Dielectric Metamaterials. *Sci. Rep.* **6**, 38487; doi: 10.1038/srep38487 (2016).

**Publisher’s note:** Springer Nature remains neutral with regard to jurisdictional claims in published maps and institutional affiliations.

## Supplementary Material

Supplementary Material

## Figures and Tables

**Figure 1:**
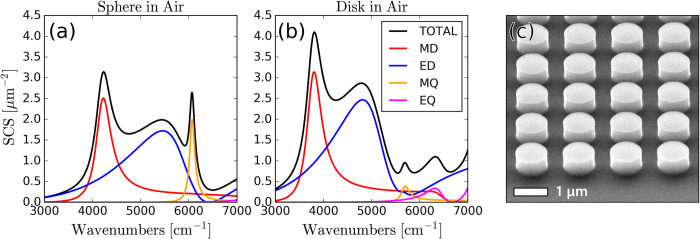
Multipolar response of dielectric resonators. The simulated multipolar decomposition of the scattering-cross-section of an (**a**) individual Silicon sphere with diameter d = 660 nm in air, and (**b**) an individual Silicon disk with height h = 660 nm and diameter d = 660 nm in air. Simulation details are in the appendices. (**c**) SEM image of one of the fabricated Silicon disk arrays.
