# Supplementary material for: Designing Multipolar Resonances in Dielectric Metamaterials
Source: Sci Rep. 2016 Dec 8;6:38487. doi: 10.1038/srep38487 (PMC5144073; doi:10.1038/srep38487)
Supplement: Supplementary Material [file srep38487-s1.doc]

**Supplementary Material:**

**Designing Multipolar Resonances in Dielectric Metamaterials**

Nikita A. Butakov and Jon A. Schuller

*University of California, Santa Barbara, Department of Electrical & Computer Engineering*

*e-mail address: schuller@ece.ucsb.edu*

**Experimental Measurement Details**

The reflection and transmission spectra of the Silicon disk resonators were measured with a Bruker Vertex FTIR and Hyperion Microscope. Simplified diagrams of the optical path in the reflection and transmission configurations are show in Figure S1. Individual isolated resonators were identified and focused using visible light in the microscope. The infrared spectra were then measured with the MCT detector.


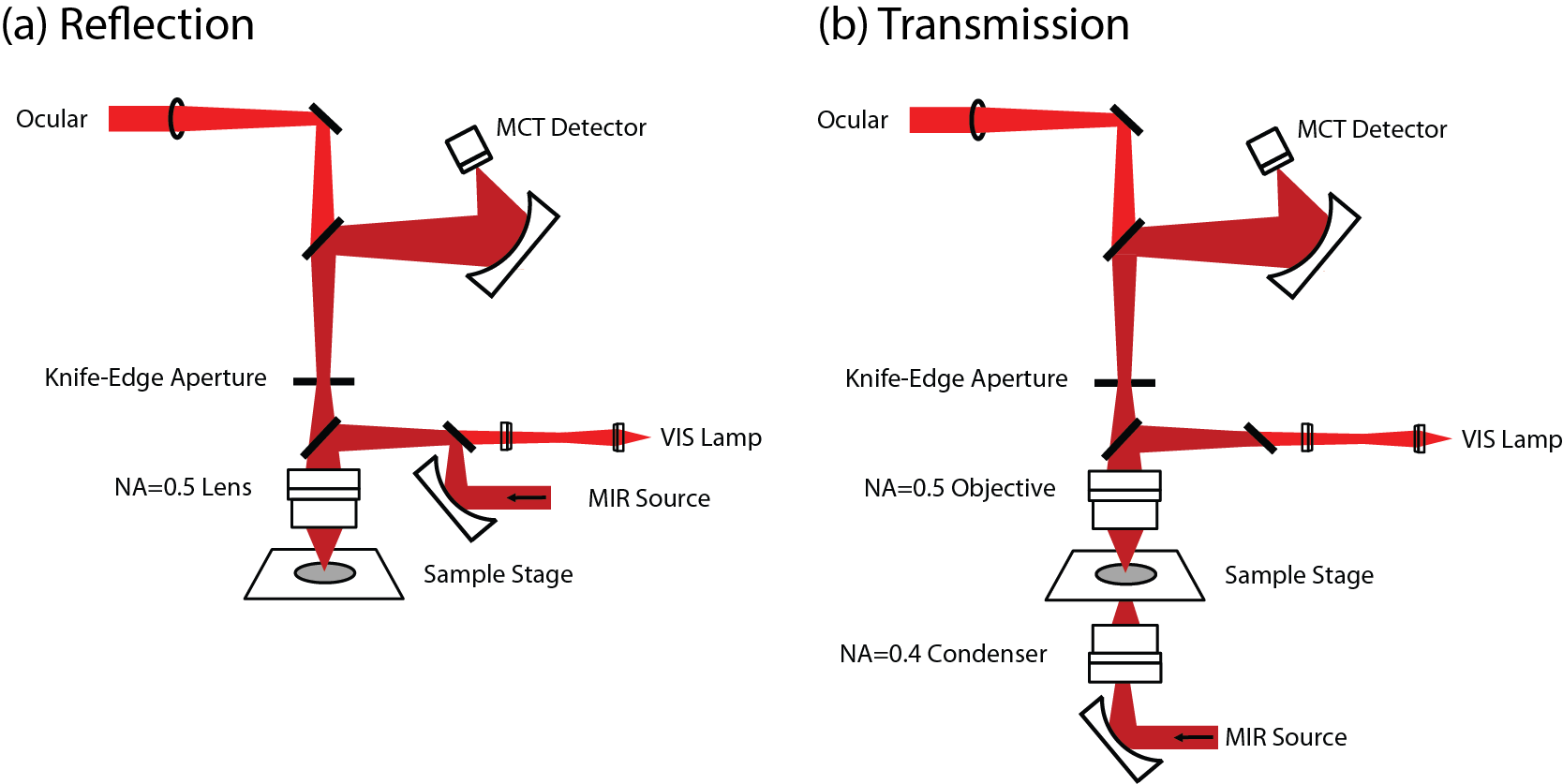


Figure S1 – **Experiment Optical Diagram.** A simplified optical path diagram of the experiment in the **(a)** reflection, and **(b)** transmission configurations.

**Experimental Spectra**

In Figure S2 we show line plots of the experimentally measured transmission and reflection of Silicon resonators on quartz and Silicon substrates respectively. Because we are essentially measuring losses compared to a highly reflecting/transmitting background, peaks in the scattering-cross-section appear as dips in the FTIR spectra. At low aspect-ratios (Figure S2ad) only a single broad peak attributable to the MD mode is visible at high frequencies. At aspect-ratios close to unity (Figure S2be), the MD and ED mode are observable. On the quartz substrate, the ED appears as a shoulder to the MD peak, and the MQ peak is visible as a small peak beyond the ED. On the silicon substrate, the ED mode is a distinct peak, although due to the much weaker signal strength noise is substantially higher. At a higher aspect ratio (Figure S2cf) the MQ mode appears as a prominent, narrow resonance on the quartz substrate. On the Silicon substrate, this peak is also strong and prominent.


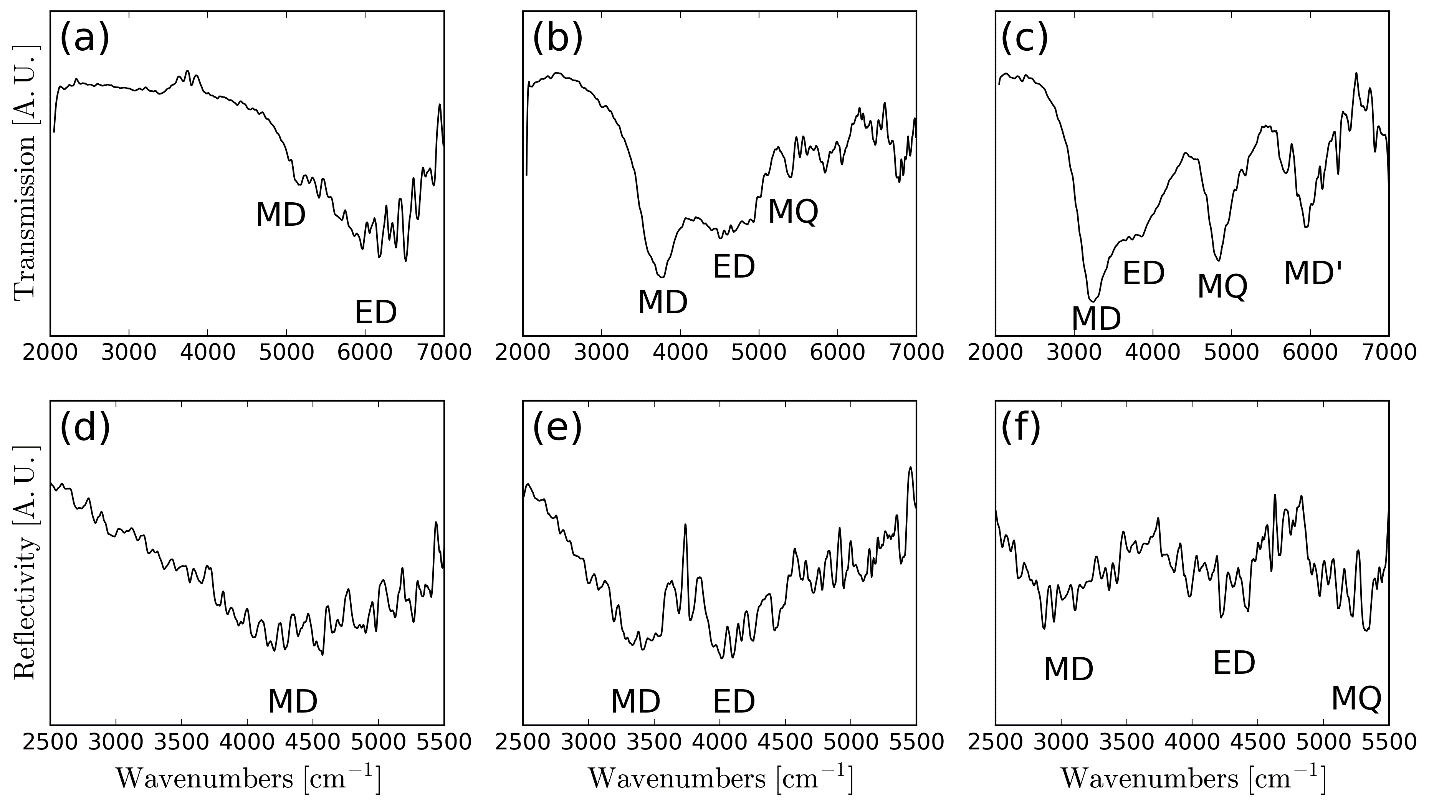
Figure S2 – **FTIR Spectra of Individual Resonators.** The measured reflection spectra of a Silicon disk on a Silicon substrate with height h=660 nm and **(a)** aspect ratio AR=0.57, **(b)** aspect ratio AR=0.97, and **(c)** aspect ratio AR=1.24. The measured transmission spectra of a Silicon disk on a Quartz substrate with height h=660 nm and **(d)** aspect ratio AR=0.57, **(e)** aspect ratio AR=1.02, and **(f)** aspect ratio AR=1.28.

In Figure S3 we show line plots of the experimentally measured transmission and reflection of an array of Silicon resonators on quartz and Silicon substrates respectively. Compared to individual resonators, the spectra have a much clearly observable resonances due to the higher signal-to-noise ratio, but a similar modal structure. The primary differences is that for resonators on the quartz substrate, the ED mode appears as a very prominent and narrow peak in the arrays, whereas it only appeared as a weak shoulder in the individual resonators.


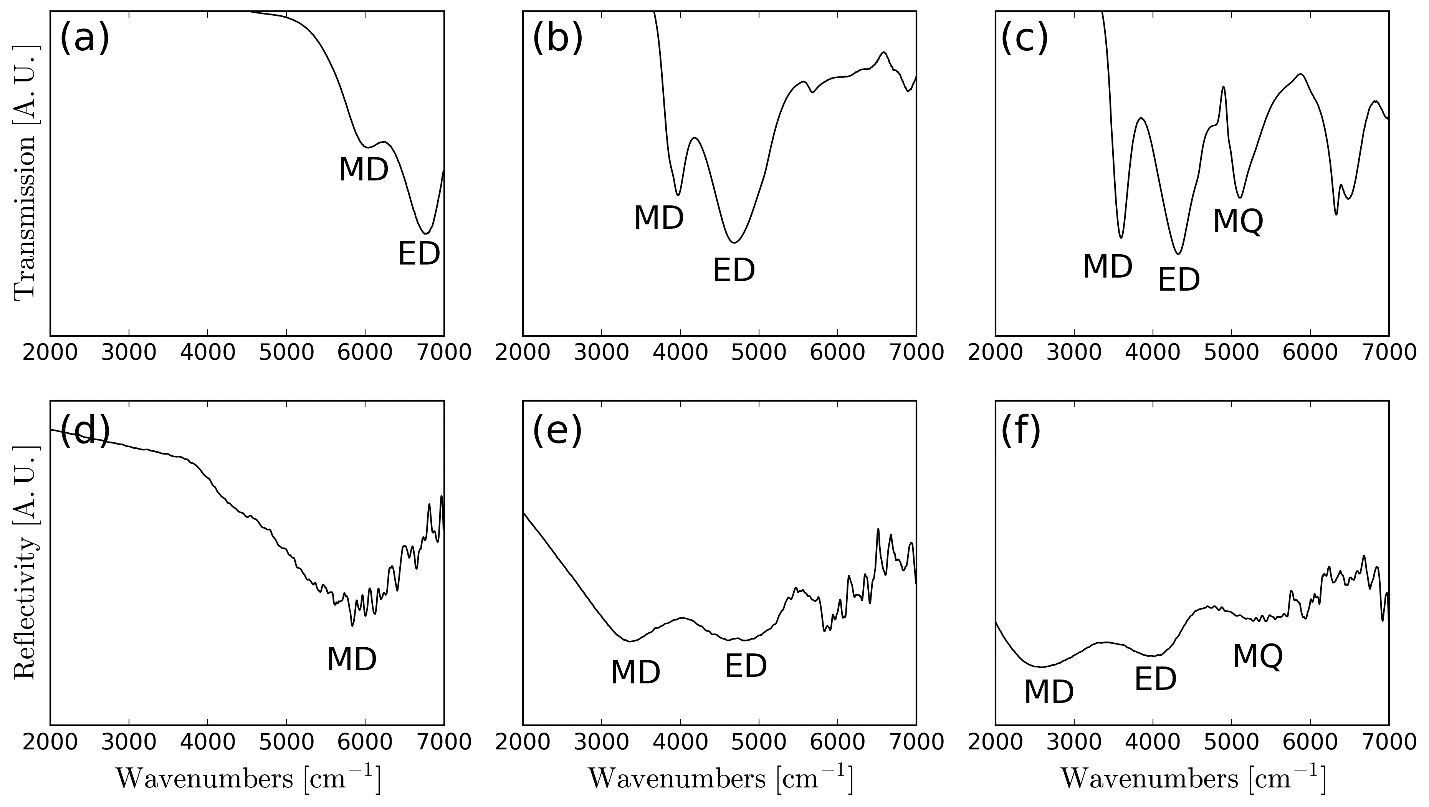
Figure S3 – **FTIR Spectra of Resonator Arrays.** The measured transmission spectra of an array of Silicon disk on a Quartz substrate with height h=660 nm and **(a)** aspect ratio AR=0.58, **(b)** aspect ratio AR=0.95, and **(c)** aspect ratio AR=1.35. The measured reflection spectra of an array of Silicon disks on a Silicon substrate with height h=660 nm and **(d)** aspect ratio AR=0.58, **(e)** aspect ratio AR=1.06, and **(f)** aspect ratio AR=1.24.
